# The heat shock factor gene family in *Salix suchowensis*: a genome-wide survey and expression profiling during development and abiotic stresses

**DOI:** 10.3389/fpls.2015.00748

**Published:** 2015-09-16

**Authors:** Jin Zhang, Yu Li, Hui-Xia Jia, Jian-Bo Li, Juan Huang, Meng-Zhu Lu, Jian-Jun Hu

**Affiliations:** ^1^State Key Laboratory of Tree Genetics and Breeding, Key Laboratory of Tree Breeding and Cultivation of the State Forestry Administration, Research Institute of Forestry, Chinese Academy of ForestryBeijing, China; ^2^Co-Innovation Center for Sustainable Forestry in Southern China, Nanjing Forestry UniversityNanjing, China

**Keywords:** abiotic stresses, gene expression, gene family, *Hsf*, *Salix suchowensis*, transcription factor

## Abstract

Heat shock transcription factors (Hsfs), which act as important transcriptional regulatory proteins, play crucial roles in plant developmental processes, and stress responses. Recently, the genome of the shrub willow *Salix suchowensis* was fully sequenced. In this study, a total of 27 non-redundant *Hsf* genes were identified from the *S. suchowensis* genome. Phylogenetic analysis revealed that the members of the *SsuHsf* family can be divided into three groups (class A, B, and C) based on their structural characteristics. Promoter analysis indicated that the *SsuHsfs* promoters included various *cis*-acting elements related to hormone and/or stress responses. Furthermore, the expression profiles of 27 *SsuHsfs* were analyzed in different tissues and under various stresses (heat, drought, salt, and ABA treatment) using RT-PCR. The results demonstrated that the *SsuHsfs* were involved in abiotic stress responses. Our results contribute to a better understanding of the complexity of the *SsuHsf* gene family, and will facilitate functional characterization in future studies.

## Introduction

As sessile organisms, plants constantly experience complex, and variable stresses in their natural environment. Therefore, plants have evolved a series of protective mechanisms for survival and reproduction. Among these protective mechanisms, the heat shock response (HSR) is a conserved cellular defense mechanism. It can be activated by a variety of cytotoxic stimuli and promotes the rapid expression of heat shock proteins (Hsps) (Morimoto et al., 1994; Schöffl et al., 1998). Hsps play crucial roles in protein folding and unfolding, the assembly of protein complexes, and protecting cells against stress (Zhang et al., 2013).

As the key regulators of Hsps, heat shock transcription factors (Hsfs) act in the upstream signal transduction pathway to activate genes in response to various abiotic/biotic stresses (Nover et al., 2001). Under normal conditions, Hsfs are blocked by molecular chaperones and maintained in a monomeric form. When exposed to stress conditions, such as heat stress, Hsfs trimerize into an active form through oligomerization domains. To promote the expression of Hsf-responsive genes, Hsfs bind to heat shock elements (HSEs), which are characterized by the conserved motif “nGAAnnTTCn,” in the promoter region (Bienz and Pelham, 1987).

The structure of Hsfs is modular, including a conserved DNA binding domain (DBD) in the N-terminus and an activation domain (AHA) in the C-terminus. The DBD is the common core structure in Hsfs, and is composed of a helix-turn-helix motif and an adjacent hydrophobic heptad repeat oligomerization domain (HR-A/B) (Nover et al., 2001). Other Hsf functional modules include a nuclear localization signal (NLS) and nuclear export signal (NES) (Kotak et al., 2004). Based on their structural characteristics, plant Hsfs can be grouped into three conserved classes (Nover et al., 2001). Among the three classes (A, B, and C), only class A members contain the AHA domain exclusively.

Compared with other eukaryotes that have 1–3 Hsfs, the plant *Hsf* family shows striking multiplicity, with more than 20 members (Von Koskull-Döring et al., 2007). As more and more whole genomic sequences of plant organisms have been released, the *Hsf* family has been analyzed extensively in many plant species (Guo et al., 2008; Lin et al., 2011; Giorno et al., 2012; Zhang et al., 2015).

Recently, willows (genus *Salix*) have become a focus of research as a potential source of sustainable and renewable biomass for bioenergy and biofuel (Hanley and Karp, 2013). *Salix suchowensis* is a native shrub willow species distributed in the north of China. It has a much smaller body size and relatively shorter juvenile period in comparison with many other tree species. The full genome sequence of *S. suchowensis* has now been published (Dai et al., 2014), which makes it possible to identify the willow *Hsf* gene family and analyze its evolutionary history in this bioenergy plant. Hsfs have been implicated in different aspects of plant life including developmental processes and abiotic/biotic stress tolerance (Kotak et al., 2007; Giorno et al., 2010). Therefore, the *Hsf* family represents a critical class of transcriptional factors to investigate. Here, we identified 27 genes encoding Hsf proteins in the *S. suchowensis* genome. To analyze the functions of the different members of this family, the expression patterns of all *SsuHsf* genes were investigated in various organs/tissues and under various abiotic stresses. These results provide a foundation for functional studies of the *SsuHsfs* in the future.

## Materials and methods

### Identification and classification of Hsfs in *S. suchowensis*

Sequencing of the *S. suchowensis* genome was completed recently, and filtered protein and coding sequences have also become available (http://115.29.234.170/cgi-bin/gbrowse/gbrowse/Ssuchowensis4/) (Dai et al., 2014). Initially, the Hsf protein sequences of *Arabidopsis thaliana* (Hübel and Schöffl, 1994) and *Populus trichocarpa* (Zhang et al., 2015) were used as queries to perform a BLASTP search against the *S. suchowensis* genome. Additionally, the Hsf domain numbered PF00447 obtained from the Pfam database (Punta et al., 2012) was used as a query to identify all possible homologs in *S. suchowensis* using BLASTP. Furthermore, the candidate sequences were analyzed in the Pfam database. The SMART program (Letunic et al., 2012) was used to detect the Hsf-type DBD domain and the coiled-coil structure.

### Phylogenetic analysis, gene structure, and domain prediction

Alignments of the full SsuHsf proteins were performed using Clustal X 2.1 (Larkin et al., 2007). Phylogenetic trees were constructed by the neighbor-joining (NJ) method in MEGA (version 5.0) (Tamura et al., 2011) with bootstrap values from 1000 replicates indicated at each node. To identify signature domains, the SsuHsf protein sequences were compared with the Hsf proteins of *A. thaliana* and *P. trichocarpa*. We named the SsuHsfs based on the subfamily classification and their phylogenetic relationships with the AtHsfs and PtHsfs. For example, the three SsuHsf members in Class A1 were named SsuHsf-A1a, SsuHsf-A1b, and SsuHsf-A1c. The pairwise comparison of Hsf amino acids was performed using MEGA (version 5.0) (Tamura et al., 2011).

The exon and intron structures were examined using the Gene Structure Display Server (GSDS) (Hu et al., 2014) by aligning the cDNA sequences with the corresponding genomic DNA sequences. The domain analysis programs MARCOIL (Delorenzi and Speed, 2002), PredictNLS (Cokol et al., 2000), and NetNES (La Cour et al., 2004) were used to predict the coiled-coil domain, NLS, and NES, respectively. In addition, the conserved motifs were defined by MEME (Bailey et al., 2009).

### *In silico* analysis of regulatory elements in the promoter regions of *SsuHsf* genes

The elements in the promoter fragments of the *SsuHsf* genes (1500 bp upstream of the translation initiation sites) were identified using the program PlantCARE online (Lescot et al., 2002).

### Plant growth conditions and treatments

Four-week-old seedlings of *S. suchowensis* clones were grown in a growth chamber under long-day conditions (16 h light/8 h dark) at 23°C. Various tissues, including the shoot tip (ST), young leaf (YL), mature leaf (ML), primary stem (PS), secondary stem (SS), root (R), and female catkin (FC) were collected from the *S. suchowensis* seedlings. For abiotic stress and hormone treatments, the seedlings were treated with 37°C (for heat stress), 20% polyethylene glycol (PEG, for drought stress), 150 mM NaCl (for salt stress), or 100 μM abscisic acid (ABA). The dosages of the abiotic stresses and hormone treatment were determined based on treatments in poplar (Shao et al., 2011; Zhang et al., 2015), and were confirmed by preliminary experiments in *S. suchowensis*. During the treatments, four time points (0, 1, 6, and 24 h) were selected for sample collection. The samples were harvested, frozen immediately in liquid nitrogen, and stored at −80°C for further analysis. Three biological replicates were performed using three completely separate sets of RNA samples from different sets of tissues for both tissue-specific experiments and stress experiments.

### RNA isolation and RT-PCR

Total RNA was extracted using the RNeasy Plant Mini Kit (Qiagen) according to the instructions. First-strand cDNA synthesis was carried out with ~2 μg RNA using the SuperScript III reverse transcription kit (Invitrogen) according to the manufacturer's procedure. Gene specific primers with melting temperatures of 58–62°C and amplicon lengths of 150–260 bp were designed using the Primer3 software (http://frodo.wi.mit.edu/primer3/input.htm). The semi-quantitative RT-PCRs were performed as follows: a pre-cycling step of 94°C for 5 min, followed by 35 (for *SsuHsf-A6a, -A6b, -A9, -B3, -B4a, -B5a*) or 30 (for other *SsuHsfs* and the internal control *SsuActin*) cycles of 94°C for 30 s, 58°C for 30 s, and 72°C for 45 s, and then a final extension at 72°C for 5 min. The 20 μl reaction system contained 10 μl Takara Premix Taq™ (Takara, Dalian, China), 1 μl of cDNA template, 1 μl of each primer, and 7 μl of ddH_2_O. The PCR products (10 μl) were electrophoresed in a 1.5% agarose gel. The *SsuActin* gene was used as an internal control. For quantitation of PCR products, the ImageJ program (NIH Image, Bethesda, MD, USA) was used to calculate relative units to indicate the fold difference between stress treatments and the control after normalization with *SsuActin*. All experiments were repeated at least three times with similar results. The fold change values were log2 transformed and the average value from three replicates were used to generate a heat map.

## Results

### Genome-wide identification and phylogenetic analysis of the *Hsf* gene family in *S. suchowensis*

To identify *Hsf* genes in *S. suchowensis*, we performed a BLASTP search against the *S. suchowensis* genome using Hsf protein sequences from *Arabidopsis* and *Populus* as queries. After removing the incomplete sequences lacking the DBD domain and/or the other functional domains, 27 non-redundant SsuHsf proteins were identified and described (Table 1). The *SsuHsfs* were distributed across 25 scaffolds of the willow genome, and two *Hsf* genes each were detected on scaffolds 10 and 25 (Table 1).

**Table 1:** **The ***Hsf*** genes identified from the ***S. suchowensis*****.

**Gene name**	**Transcrit ID**	**Map position (bp)**	**Length (aa)**	**MW (kDa)/pI**	***A. thaliana* ortholog locus**	***P. trichocarpa* ortholog locus**
*SsuHsf-A1a*	willow_GLEAN_10025706	scaffold25:1497258-1497907(+)	497	54.3/4.68	AT1G32330.1	Potri.003G095000.1
*SsuHsf-A1b*	willow_GLEAN_10004399	scaffold185:94000-96478(+)	476	52.8/5.39	AT5G16820.1	Potri.013G079800.1
*SsuHsf-A1c*	willow_GLEAN_10014876	scaffold79:510224-511723(+)	508	55.7/4.83	AT1G32330.1	Potri.001G138900.1
*SsuHsf-A2*	willow_GLEAN_10026187	scaffold19:533642-535574(+)	374	42/4.85	AT2G26150.1	Potri.006G226800.1
*SsuHsf-A3*	willow_GLEAN_10026517	scaffold18:2169295-2172020(+)	519	57.9/4.85	AT5G03720.1	Potri.006G115700.1
*SsuHsf-A4a*	willow_GLEAN_10005943	scaffold181:32748-37435(+)	406	45.9/5.09	AT4G18880.1	Potri.011G071700.1
*SsuHsf-A4b*	willow_GLEAN_10018721	scaffold69:816974-819687(−)	444	50.8/5.65	AT4G18880.1	Potri.014G141400.1
*SsuHsf-A4c*	willow_GLEAN_10017256	scaffold71:700318-701359(−)	407	46.5/5.42	AT4G18880.1	Potri.004G062300.1
*SsuHsf-A5*	willow_GLEAN_10019246	scaffold66:357257-358822(+)	489	54.7/6.01	AT4G13980.1	Potri.001G320900.1
*SsuHsf-A6a*	willow_GLEAN_10021781	scaffold41:90931-92291(−)	362	41.6/4.98	AT3G22830.1	Potri.010G082000.1
*SsuHsf-A6b*	willow_GLEAN_10003707	scaffold205:80301-82019(−)	368	42/5.05	AT3G22830.1	Potri.008G157600.1
*SsuHsf-A7a*	willow_GLEAN_10001664	scaffold01442:1030-2689(+)	361	40.9/6.64	AT3G22830.1	Potri.005G214800.1
*SsuHsf-A7b*	willow_GLEAN_10022356	scaffold25:386922-388292(+)	360	41.1/5.51	AT3G22830.1	Potri.002G048200.1
*SsuHsf-A8a*	willow_GLEAN_10010667	scaffold143:390791-392136(+)	402	46.1/4.89	AT1G67970.1	Potri.010G104300.1
*SsuHsf-A8b*	willow_GLEAN_10021820	scaffold37:1365061-1368951(−)	391	44.6/4.74	AT1G67970.1	Potri.010G104300.1
*SsuHsf-A9*	willow_GLEAN_10020699	scaffold56:263821-265104(+)	555	61.7/4.78	AT2G26150.1	Potri.006G148200.1
*SsuHsf-B1*	willow_GLEAN_10004276	scaffold10:3329791-3332899(+)	275	30/5.1	AT4G36990.1	Potri.007G043800.1
*SsuHsf-B2a*	willow_GLEAN_10009738	scaffold10:2825166-2826872(+)	314	34.8/5.41	AT5G62020.1	Potri.015G141100.1
*SsuHsf-B2b*	willow_GLEAN_10004530	scaffold183:89947-91189(+)	352	37.9/4.98	AT4G11660.1	Potri.001G108100.1
*SsuHsf-B3*	willow_GLEAN_10014050	scaffold8:1488988-1490578(+)	204	23.8/7.66	AT2G41690.1	Potri.016G056500.1
*SsuHsf-B4a*	willow_GLEAN_10009316	scaffold3:4044788-4049120(−)	189	21.5/5.17	AT1G46264.1	Potri.002G124800.1
*SsuHsf-B4b*	willow_GLEAN_10024472	scaffold13:1822238-1824250(−)	271	31.2/6.9	AT1G46264.1	Potri.009G068000.1
*SsuHsf-B4c*	willow_GLEAN_10004301	scaffold192:153539-154003(+)	377	42.1/8.64	AT1G46264.1	Potri.014G027100.1
*SsuHsf-B4d*	willow_GLEAN_10011830	scaffold85:641587-647118(−)	270	30.9/6.59	AT1G46264.1	Potri.001G273700.1
*SsuHsf-B5a*	willow_GLEAN_10017386	scaffold1:81191-83601(+)	180	20.4/9.77	AT4G17750.1	Potri.004G042600.1
*SsuHsf-B5b*	willow_GLEAN_10010880	scaffold2:3463989-3465649(−)	203	23.2/7.77	AT1G32330.1	Potri.011G051600.1
*SsuHsf-C1*	willow_GLEAN_10010554	scaffold177:244037-245200(−)	316	34.9/5.3	AT3G24520.1	Potri.T137400.1

**Figure 1:**
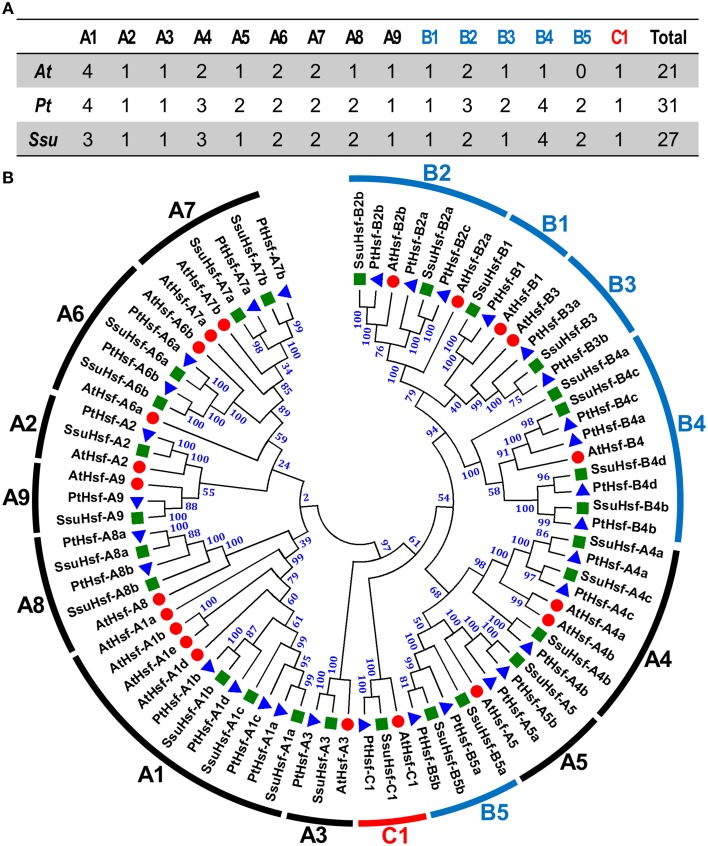
**Hsf family members (A) and their phylogenetic relationships (B) from ***S. suchowensis***, ***P. trichocarpa***, and ***A. thaliana*****. Multiple alignment was performed using Clustal X 2.1. Phylogenetic tree was constructed by the neighbor-joining (NJ) method with 1000 bootstrap replicates. Bootstrap support values are indicated on each node. The three major groups are marked with different colors. The complete sequences of identified Hsfs are listed in Table S1. Hsfs in *S. suchowensis, P. trichocarpa*, and *A. thaliana* were marked with green squares, blue triangles, and red circles, respectively.

Based on the multiple sequence alignment of the DBD and HR-A/B, the 27 SsuHsfs were grouped into Class A (16 genes), Class B (10 genes), and Class C (one gene) (Table 1 and Figure 1A). The SsuHsf protein lengths ranged from 180 to 555 amino acids, and their predicted isoelectric points ranged from 4.68 to 9.77 (Table 1).

To investigate the evolutionary relationships of the Hsfs, an unrooted phylogenetic tree was generated using the full length protein sequences of the 27 *S. suchowensis* Hsfs (SsuHsfs), 31 *P. trichocarpa* Hsfs (PtHsfs), and 21 *A. thaliana* Hsfs (AtHsfs) (Table 2). As shown in Figure 1B, the Hsfs of the three species were distinctly classified into three classes (A, B, and C). The Class C Hsfs from the three plant species constituted a distinct clade. The size of the Class A1, A5, B2, and B3 SsuHsfs were smaller than those in *P. trichocarpa*. We named the SsuHsfs based on the subfamily classification and their phylogenetic relationships with the AtHsfs and PtHsfs. For example, three SsuHsf members in Class A1 were named SsuHsf-A1a, SsuHsf-A1b, and SsuHsf-A1c.

**Table 2:** **Comparison of Hsf members in ***S. suchowensis, P. trichocarpa***, and ***A. thaliana*****.

	**Hsfs**		***S. suchowensis***	***P. trichocarpa***	***A. thaliana***
			**27**	**31**	**21**
Type A	A1	A1a	willow_GLEAN_10025706	Potri.003G095000.1	At4g17750.1
		A1b	willow_GLEAN_10004399	Potri.013G079800.1	At5g16820.1
		A1c	willow_GLEAN_10014876	Potri.001G138900.1	At1g32330.1
		A1d		Potri.019G050400.1	At3g02990.1
	A2	A2	willow_GLEAN_10026187	Potri.006G226800.1	At2g26150.1
	A3	A3	willow_GLEAN_10026517	Potri.006G115700.1	At5g03720.1
	A4	A4a	willow_GLEAN_10005943	Potri.011G071700.1	At4g18880.1
		A4b	willow_GLEAN_10018721	Potri.014G141400.1	At5g45710.1
		A4c	willow_GLEAN_10017256	Potri.004G062300.1	
	A5	A5a	willow_GLEAN_10019246	Potri.017G059600.1	At4g13980.1
		A5b		Potri.001G320900.1	
	A6	A6a	willow_GLEAN_10021781	Potri.010G082000.1	At5g43840.1
		A6b	willow_GLEAN_10003707	Potri.008G157600.1	At3g22830.1
	A7	A7a	willow_GLEAN_10001664	Potri.005G214800.1	At3g51910.1
		A7b	willow_GLEAN_10022356	Potri.002G048200.1	At3g63350.1
	A8	A8a	willow_GLEAN_10010667	Potri.008G136800.1	At1g67970.1
		A8b	willow_GLEAN_10021820	Potri.010G104300.1	
	A9	A9	willow_GLEAN_10020699	Potri.006G148200.1	At5g54070.1
Type B	B1	B1	willow_GLEAN_10004276	Potri.007G043800.1	At4g36990.1
	B2	B2a	willow_GLEAN_10009738	Potri.012G138900.1	At5g62020.1
		B2b	willow_GLEAN_10004530	Potri.001G108100.1	At4g11660.1
		B2c		Potri.015G141100.1	
	B3	B3a	willow_GLEAN_10014050	Potri.006G049200.1	At2g41690.1
		B3b		Potri.016G056500.1	
	B4	B4a	willow_GLEAN_10009316	Potri.002G124800.1	At1g46264.1
		B4b	willow_GLEAN_10024472	Potri.009G068000.1	
		B4c	willow_GLEAN_10004301	Potri.014G027100.1	
		B4d	willow_GLEAN_10011830	Potri.001G273700.1	
	B5	B5a	willow_GLEAN_10017386	Potri.004G042600.1	
		B5b	willow_GLEAN_10010880	Potri.011G051600.1	
Type C	C1	C1	willow_GLEAN_10010554	Potri.T137400.1	At3g24520.1

### Structural analysis of *Hsfs* in *S. suchowensis*

**Figure 2:**
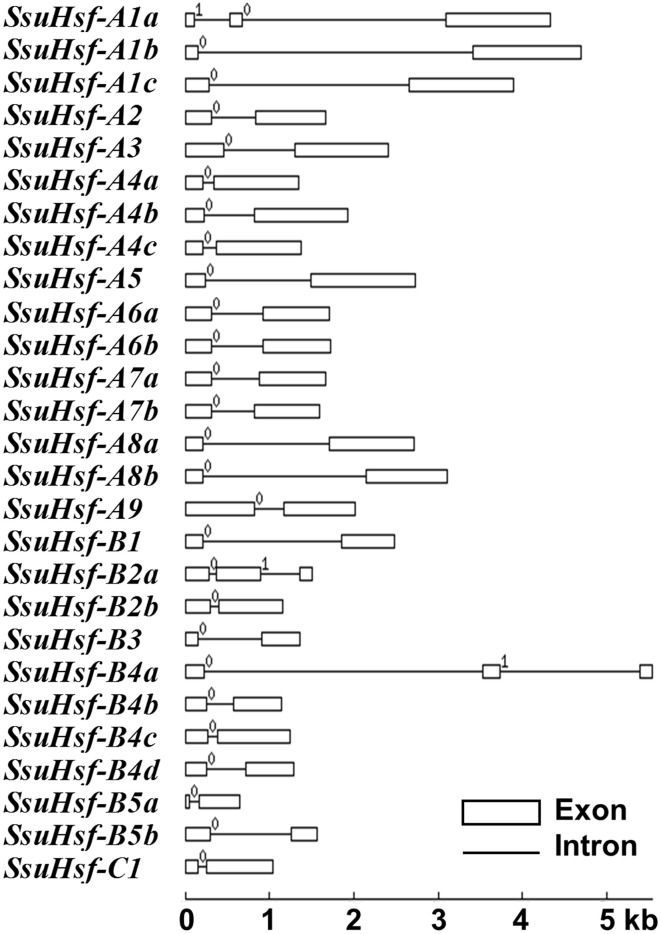
**Gene structures of ***SsuHsf*** genes**. Boxes represent exons and lines represent introns. The numbers indicate the splicing phases of the *SsuHsfs*: 0, phase 0; 1, phase 1; and 2, phase 2.

To evaluate the structural diversity of the *SsuHsf* genes, the full-length cDNA sequences were compared with the corresponding genomic DNA sequences to determine the numbers and positions of exons and introns within each gene (Figure 2). Exon/intron structural divergence within a gene family plays a critical role during evolution. In general, paralogous genes are highly conserved in gene structure and this conservation is sufficient to reveal their evolutionary relationships (Hardison, 1996). Most *SsuHsf* genes included only one intron, except for *SsuHsf-A1a, SsuHsf-B2a*, and *SsuHsf-B4a*, which included two introns. The intron phases were remarkably well-conserved among family members (Figure 2).

**Figure 3:**
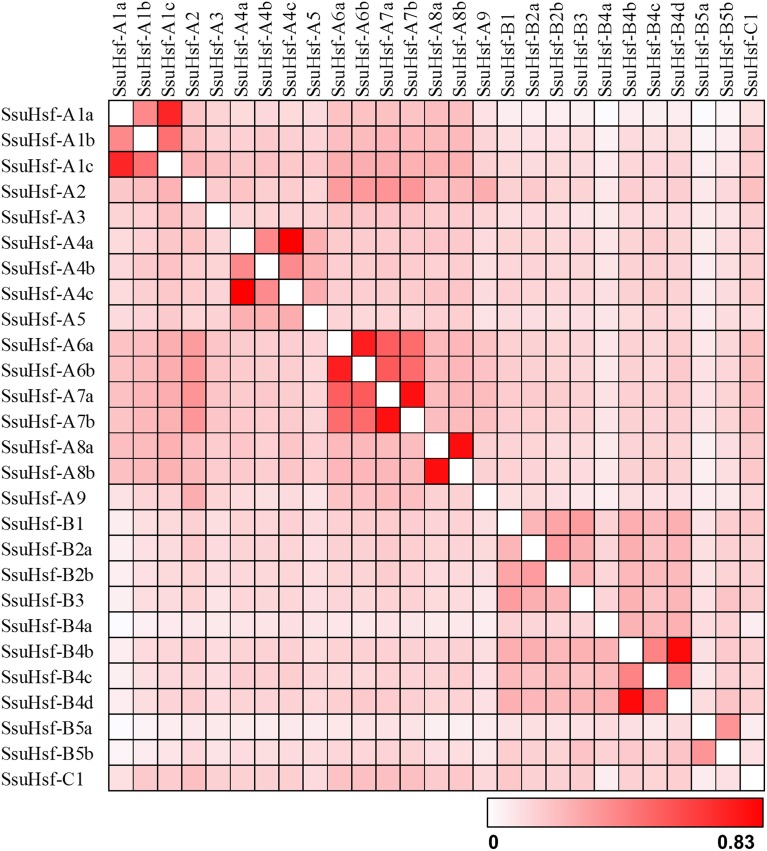
**Sequence identity of SsuHsf proteins**. Amino acid identity among SsuHsf proteins was analyzed in pairwise fashion.

The sequence conservation among SsuHsf proteins was also supported by their identity at the amino acid level (0.023–0.83, Figure 3). Six pairs of SsuHsfs (A1a-A1c, A4a-A4c, A6a-A6b, A7a-A7b, A8a-A8b, and B4b-B4d) exhibited high sequence identity. Detailed information on the identity among SsuHsf, PtHsf, AtHsf amino acid sequences is shown in Figure S1.

### Duplication of *Hsfs* in *S. suchowensis*

Based on the phylogenetic relationships and gene structures of the *SsuHsf* genes (Figures 1, 2), we found that all five *SsuHsf* paralogous gene pairs were generated by duplication events (Table 3). To verify whether Darwinian positive selection was involved in the *SsuHsf* genes' divergence after duplication, the substitution rate ratio of non-synonymous (*K*a) vs. synonymous (*K*s) substitutions was calculated for the *SsuHsf* gene pairs. In general, *K*a/*K*s ratio implies different selection types: positive selection (>1), neutral selection (= 1), or purifying selection (< 1) (Hurst, 2002). As shown in Table 3, the *K*a/*K*s ratios of all five *SsuHsf* gene pairs were less than 0.4; thus, it can be concluded that the *SsuHsf* gene family has undergone great purifying selection pressure with limited functional divergence after duplication. Notably, the average values of *K*a and *K*s in *S. suchowensis Hsf* gene pairs were larger than those in *P. trichocarpa* (*K*a was ~0.1084 in *SsuHsf* pairs and ~0.0702 in *PtHsf* pairs, *K*s was ~0.3305 in *SsuHsf* pairs and ~0.2699 in *PtHsf* pairs) (Zhang et al., 2015).

**Table 3:** **Divergence between paralogous ***SsuHsf*** gene pairs**.

**No**.	**Gene 1**	**Gene 2**	***K*a**	***K*s**	***K*a/*K*s**
1	*SsuHsf-A4a*	*SsuHsf-A4c*	0.0877	0.3092	0.2837
2	*SsuHsf-A6a*	*SsuHsf-A6b*	0.1184	0.2960	0.3999
3	*SsuHsf-A7a*	*SsuHsf-A7b*	0.1194	0.3479	0.3431
4	*SsuHsf-A8a*	*SsuHsf-A8b*	0.0998	0.3203	0.3118
5	*SsuHsf-B4b*	*SsuHsf-B4d*	0.1169	0.3789	0.3084

*Gene pairs were identified based on the phylogenetic tree (Figure 1). Ka and Ks rates are presented for each pair*.

### Conserved domains and motifs of SsuHsfs

**Figure 4:**
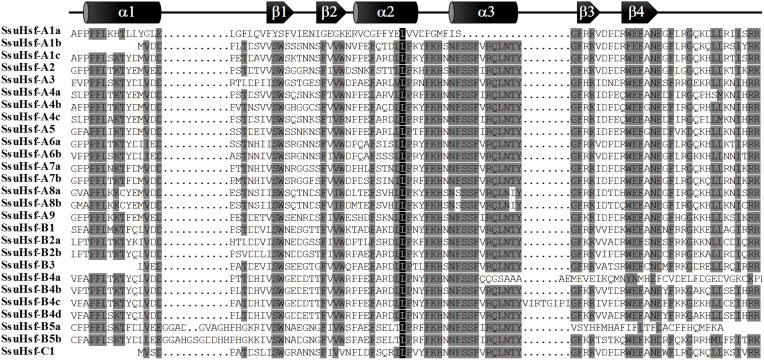
**Multiple sequence alignment of the DBD domains of the SsuHsf proteins**. The secondary structures of the DBD (α1-β1-β2-α2-α3-β3-β4) are shown above the alignment. α-helices and β-sheets were marked using cylindrical tubes and block arrows, respectively.

The modular structures of Hsfs have been studied thoroughly in some model plants (Nover et al., 2001; Scharf et al., 2012). The known information on functional domains of AtHsfs makes it possible to identify similar domains in the SsuHsfs. As shown in Table 4, five conserved domains (DBD, HR-A/B, NLS, NES, and AHA) were identified by sequence alignment and their positions in the proteins. The conserved DBD comprised three α-helices (α1–3) and four β-sheets (β1–4) (Figure 4). It has been reported that NES and NLS domains are essential for shuttling Hsfs between the nucleus and cytoplasm (Scharf et al., 2012), and the majority of the SsuHsfs showed the presence of a NES and/or NLS domain. Furthermore, AHA motifs were identified in most of the Class A SsuHsfs. However, we were unable to predict putative AHA motifs in the Class B and C proteins (Table 4).

**Table 4:** **Functional domains of SsuHsfs**.

**Gene Name**	**DBD**	**HR-A/B**	**NLS**	**NES**	**AHA1**	**AHA2**
*SsuHsf-A1a*	33–116	149–199	(229) NKKRRLKQ	(481) VEQLTEQMG	(438) SSFWYDLLVQ	
*SsuHsf-A1b*	1–83	111–158	(191) SKKRRLPR	(459) MNHLAEQME	(411) DVFWEQFLTA	
*SsuHsf-A1c*	33–126	159–207	(239) NKKRRLKQ	(491) MDQLTEQMG	(448) SSFWDDLLVQ	
*SsuHsf-A2*	40–133	159–201	(229) RR-X_8_-RKRR	(363) LVDQMGYL	(315) ETIWEELFSD	(355) DWSDDFQD
*SsuHsf-A3*	90–183	207–252	(270) ARLKQKKEQ	N.D.	(443) W-X_17_-W-X_20_-W-X_15_-W	
*SsuHsf-A4a*	10–103	128–171	(204) DRKRRL	(393) LTEQIGHL	(257) LTFWENMVHD	(342) DVFWEQFLTE
*SsuHsf-A4b*	11–104	126–178	(203) NKKRKA	(431) LAMHTGQI	(253) LKFLENFLYA	(378) DLFWQHFLTE
*SsuHsf-A4c*	10–103	129–174	(204) DRKRRL	(394) LTEQMGHL	(258) LTFWENMVHD	(343) DVFWEQFLTE
*SsuHsf-A5*	17–110	132–179	(199) RK-X_10_-KKRR	(484) MEQLSL	(438) DVFWEQFLTE	
*SsuHsf-A6a*	40–133	153–195	(234) KKKRR	(350) LVEQLGYM	(319) EAFWEDLLNE	
*SsuHsf-A6b*	41–134	162–207	(240) KKRRR	(343) LGGEGED	(325) EVFWEDLLNE	
*SsuHsf-A7a*	42–135	163–234	(231) KRKELEEALTKKRRR	(349) LAERLGYL	(327) EGFWEELLNE	
*SsuHsf-A7b*	42–135	162–228	(231) KTKELEEAMTKKRRR	(345) LAERLNYL	(323) EGFWEELLNE	
*SsuHsf-A8a*	8–101	141–175	(100) RRK	(381) TKQMGLL	(299) DGAWEQLLL	
*SsuHsf-A8b*	8–101	134–175	(100) RRK	(379) TWQMDHL	(298) DGSWEHMFL	
*SsuHsf-A9*	212–305	328–368	(296) KHLLKSIKRR	(522) LYLELEDL	nd	
*SsuHsf-B1*	6–99	148–180	(242) LFGV-X_6_-KKKR	nd	nd	
*SsuHsf-B2a*	31–124	171–215	(111) RKGKK	nd	nd	
*SsuHsf-B2b*	36–129	199–216	(116) RRGEK	nd	nd	
*SsuHsf-B3*	2–84	124–168	(171) LFGV-X_9_-RKRK	nd	nd	
*SsuHsf-B4a*	21–121	N.D.	(158) SRKAFRFNERRR	nd	nd	
*SsuHsf-B4b*	21–114	153–186	(254) LFGV-X_4_-NKR	nd	nd	
*SsuHsf-B4c*	21–122	203–235	(331) LFGV-X_4_-KKR	nd	nd	
*SsuHsf-B4d*	21–114	153–184	(253) LFGV-X_4_-NKR	nd	nd	
*SsuHsf-B5a*	68–164	N.D.	(14) KKTKKK	nd	nd	
*SsuHsf-B5b*	28–132	166–186	(120) KGRR	nd	nd	
*SsuHsf-C1*	1–83	112–137	(81) VRRKHG	nd	nd	

*nd, no motifs detectable by sequence similarity search*.

**Figure 5:**
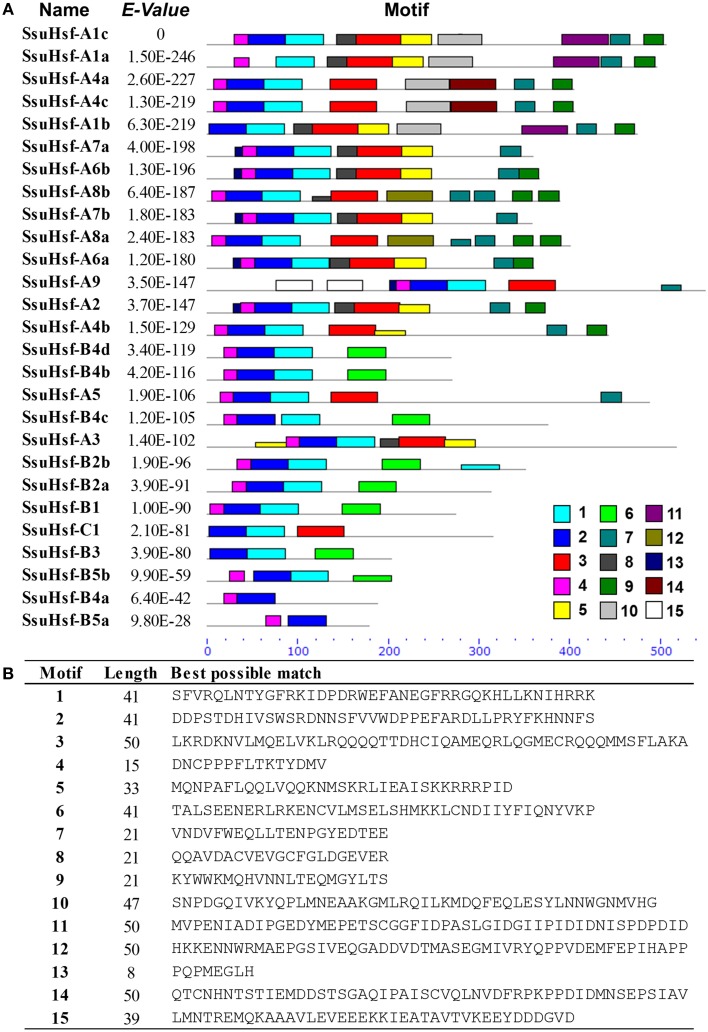
**Distribution of conserved motifs in the SsuHsf proteins**. **(A)** The motifs were identified by MEME. Different motifs are indicated by different colored numbers 1–15. **(B)** The detail motif sequences.

After searching with the MEME motif search tool, 15 consensus motifs were detected in the SsuHsfs (Figure 5). The majority of SsuHsfs possessed motifs 1, 2, and 4, which corresponded to highly conserved regions including the DBD region. Specifying the coiled-coil structure, motifs 3 and 6 were distinctly detected in all SsuHsfs. However, motif 3 only existed in the Class A and C SsuHsfs, and motif 6 was only present in Class B SsuHsfs. Motifs 5 and 9 included the NLS and NES, respectively. Furthermore, motif 7 represented the AHA motif close to the Hsf C-terminus (Figure 5 and Table 4).

### *cis*-elements in the promoter regions of *SsuHsfs*

**Figure 6:**
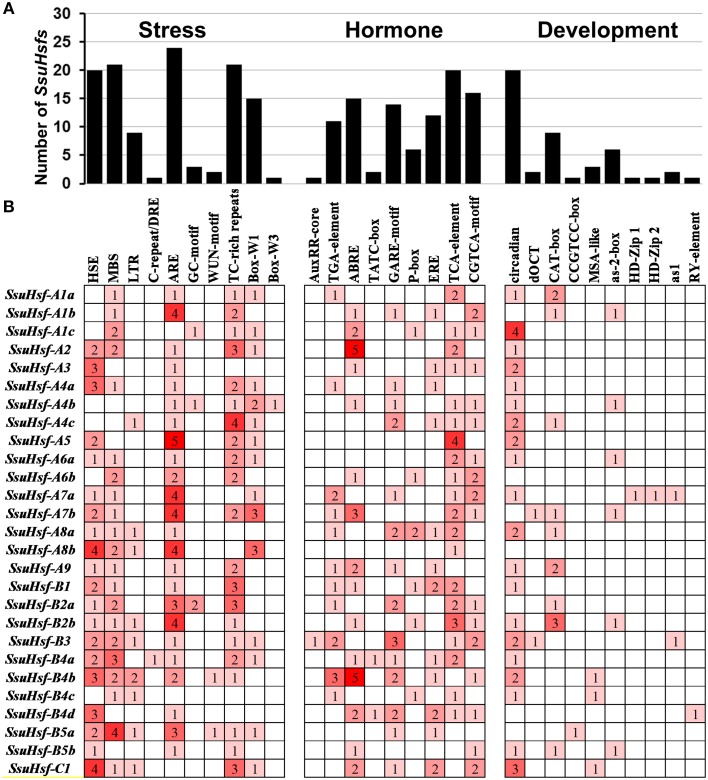
**Various ***cis***-acting elements in ***SsuHsf*** genes. (A)** The number of *SsuHsf* genes containing various *cis*-acting elements. **(B)** The number of occurrences of each *cis*-acting elements in the promoter region of each of *SsuHsf* genes. The annotation of the *cis*-elements: HSE, *cis*-acting element involved in heat stress responsiveness; MBS, MYB binding site involved in drought-inducibility; LTR, involved in low-temperature responsiveness; C-repeat/DRE, involved in cold- and dehydration-responsiveness; ARE, essential for the anaerobic induction; GC-motif, enhancer-like element involved in anoxic specific inducibility; WUN-motif, wound-responsive element; TC-rich repeats, involved in defense and stress responsiveness; Box-W1 and Box-W3, fungal elicitor responsive element; AuxRR-core and TGA-element, auxin-responsive element; ABRE, involved in the abscisic acid responsiveness; TATC-box, GARE-motif and P-box, gibberellin-responsive element; ERE, ethylene-responsive element; TCA-element, involved in salicylic acid responsiveness; CGTCA-motif, involved in the MeJA-responsiveness; circadian, involved in circadian control; dOCT and CAT-box, related to meristem expression; CCGTCC-box, related to meristem specific activation; MSA-like, involved in cell cycle regulation; as-2-box, involved in shoot-specific expression and light responsiveness; HD-Zip1, involved in differentiation of the palisade mesophyll cells; HD-Zip2, involved in the control of leaf morphology development; as1, involved in the root-specific expression; RY-element, involved in seed-specific regulation.

To identify the likely *cis*-elements of the *SsuHsfs*, the promoter regions (1.5 kb of genomic DNA sequence upstream of the translation start site) of the *SsuHsf* genes were used to search the PlantCARE database. A series of *cis*-elements involved in abiotic stress responses, phytohormone responses, and developmental processes were identified. As shown in Figure 6, the SA-responsive element (TCA-element), the MeJA-responsive element (CGTCA-motif), and the ABA-responsive element (ABRE) were found in the promoters of 20, 16, and 15 *SsuHsf* genes, respectively. All three were present in the promoter regions of seven genes. The HSE was found in the promoters of 20 *SsuHsf* genes. The anaerobic induction element (ARE), defense and stress responsive element (TC-rich), and MYB binding sites involved in drought-inducibility (MBS) were found in 24, 21, and 21 *SsuHsf* gene promoters, respectively. Additionally, the circadian control element (circadian) was found in the promoters of 20 *SsuHsfs*. Notably, two leaf development related *cis*-elements (HD-Zip1 and HD-Zip2) were found in the *SsuHsf-A7a* promoter. These results indicated that the *SsuHsfs* might be involved in the transcriptional control of hormone and stress responses and developmental processes.

### Expression profiles of *SsuHsf* genes in various tissues

**Figure 7:**
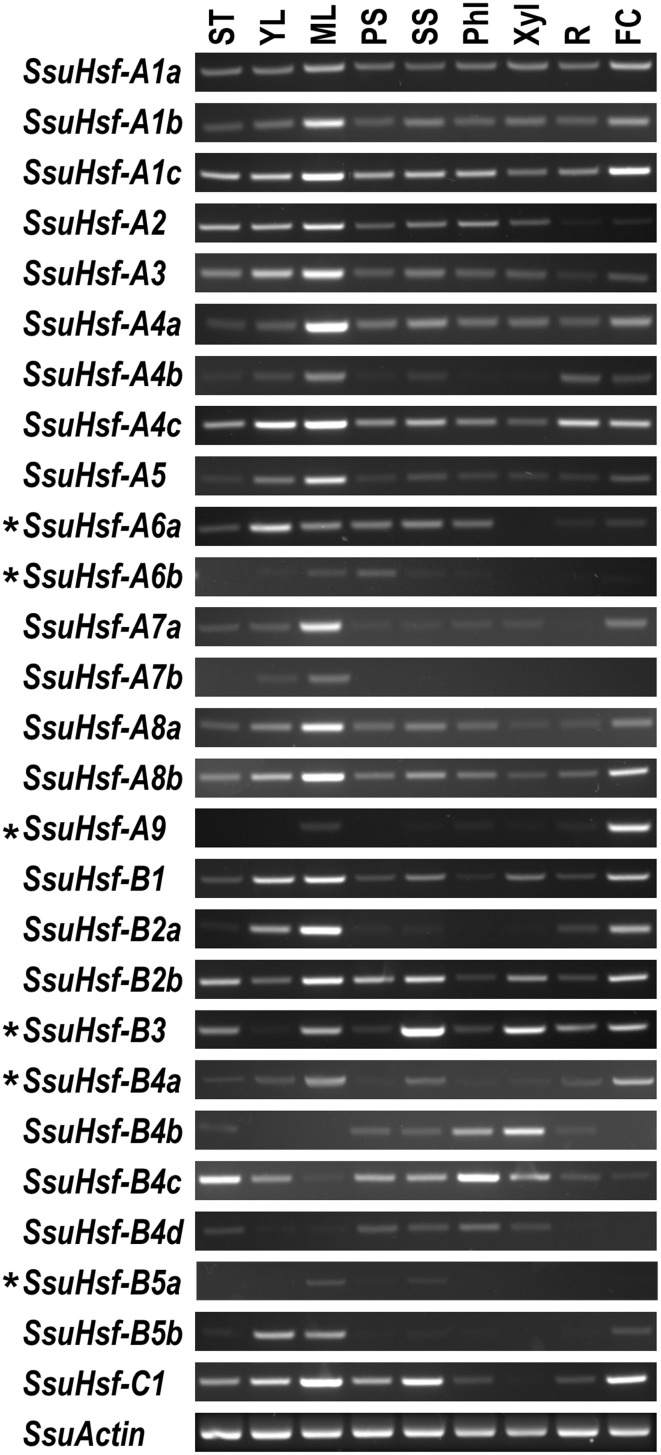
**Expression analyses of ***SsuHsfs*** in different tissues**. The expression of 27 *SsuHsfs* in shoot tip (ST), young leaf (YL), mature leaf (ML), primary stem (PS), secondary stem (SS), phloem (Phl), xylem (Xyl), root (R), and female catkin (FC) from *S. suchowensis*. The amplification cycle used was 35 for six asterisk (^*^) labeled genes and 30 for all the other *SsuHsfs* and the *SsuActin* reference control. One experiment representative for three biological replicate experiments was shown in here. Primers used for RT-PCR are listed in Table S2.

To identify the spatial and temporal expression patterns of the *SsuHsfs*, RT-PCR was performed on the 27 *SsuHsfs* in nine different tissues of *S. suchowensis*: the shoot tip (ST), young leaf (YL), mature leaf (ML), primary stem (PS), secondary stem (SS), phloem (Phl), xylem (Xyl), root (R), and female catkin (FC). Most *SsuHsfs* showed distinct tissue expression patterns. As shown in Figure 7, some genes had tissue-specific expression patterns; for example, *SsuHsf-B3* was highly expressed in the secondary stem and xylem, *SsuHsf-B4c* was highly expressed in the shoot tip and phloem, and *SsuHsf-A7a* was highly expressed in the mature leaf. Interestingly, *SsuHsf-A9* was specifically expressed in the female catkin.

Among the five pairs of *SsuHsf* paralogs, one pair (*SsuHsf-A8a*/*A8b*) exhibited similar expression patterns in the analyzed tissues, while the other four pairs showed different tissue expression patterns to some degree (Figure 7).

### Expression analysis of *SsuHsf* genes in response to various treatments

**Figure 8:**
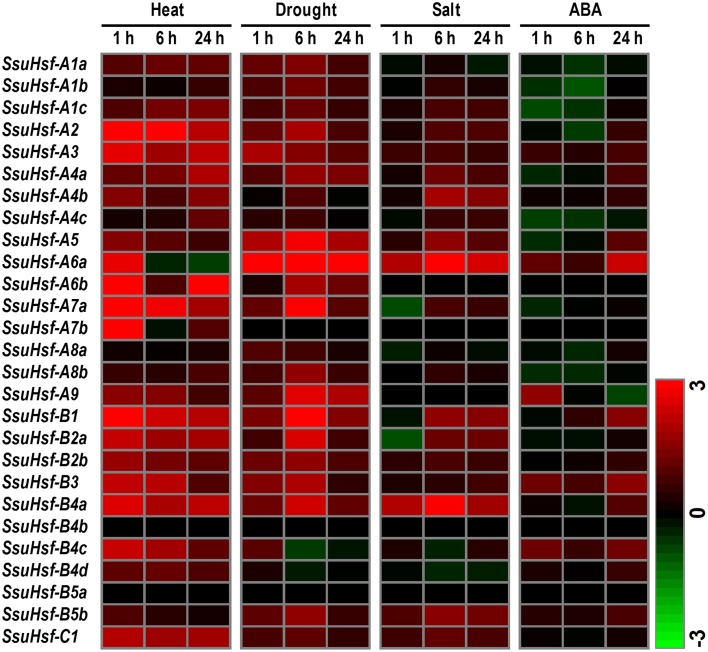
**Expression analyses of ***SsuHsfs*** under abiotic stresses**. Heat map representation for the expression patterns of 27 *SsuHsf* genes after treated for 1, 6, or 24 h under heat (37°C), drought (20% PEG), salt (150 mM NaCl), or 100 μM ABA. The expression levels of genes were determined using RT-PCR. The different colors correspond to log2 transformed values compared with control (0 h). Green indicates down-regulation and red represents up-regulation. The data were generated by averaging the fold change from each of the three biological replicate experiments. Details of the expression data are listed in Table S3.

To determine the potential roles of the *SsuHsf* genes in plant responses to various environmental stresses, RT-PCR was performed on the 27 *SsuHsf* genes in the leaves of *S. suchowensis* seedlings exposed to heat, drought, salt, and ABA treatments. Overall, except for *SsuHsf-B4b* and *SsuHsf-B5a*, the transcript levels of all of the *SsuHsf* genes responded to at least one treatment (Figure 8). Among them, 10 *SsuHsfs* (*A1c, A2, A3, A5, A6a, B1, B2a, B2b, B4a*, and *C1*) were significantly induced by heat, drought, and salt stress, and five *SsuHsfs* (*A4b, A7a, A9, B3*, and *B5b*) responded to two treatments (Figure 8). This indicated that these genes might be nodes of convergence for different stress response pathways. In response to heat, 24 of the 27 *SsuHsf* genes were induced. Notably, three members including *A6b, A9*, and *B4d* showed no or low expression in leaves under normal growth conditions (Figure 7), but were strongly up-regulated during the heat stress treatment (Figure 8). In addition, most of the *SsuHsfs* (*A2, A3, A6a, A6b, A7a, A7b, B1, B2a, B2b, B3, B4a, B4c*, and *C1*) showed immediate transcript accumulation at 1 h in the 37°C treatment.

## Discussion

### Characterization of the *S. suchowensis Hsf* gene family

A total of 27 non-redundant *Hsfs* were identified based on the recently released *S. suchowensis* genome (Dai et al., 2014). The size of the *Hsf* family in *S. suchowensis* is smaller than in *P. trichocarpa*, which is consistent with the genome sizes of these two species (~425 Mb in *S. suchowensis* and ~485 Mb in *P. trichocarpa*) (Dai et al., 2014). Phylogenetic analyses of the Hsfs in *S. suchowensis, P. trichocarpa*, and *A. thaliana* indicated that the SsuHsfs are correspond more closely with the PtHsfs than the AtHsfs, consistent with the evolutionary relationships among the three species. All three *Hsf* classes (Classes A, B, and C) were identified in all three species, implying that the *Hsf* genes originated prior to the divergence of these species.

During evolution, gene duplication plays a critical role in the expansion of gene families (Maere et al., 2005). Among the 27 *SsuHsfs*, five pairs of *SsuHsf* gene paralogs were identified, and the members in each pair were distributed on different scaffolds. This suggests the *SsuHsf* gene family expansion originated from large segmental duplications. It has been reported that more than 90% of the increased regulatory genes in *Arabidopsis* were generated by genome duplication events in the last ~150 million years (Maere et al., 2005). Individual gene family expansion follows this rule similarly. Our results suggest that *SsuHsf* gene pairs have a higher substitution rate than those in *P. trichocarpa*. The great differences in evolutionary rates between the two species are correlated with their flowering habits: the early-flowering species (*S. suchowensis* flowers within 2 years) has faster substitution rates than the long-generation one (Dai et al., 2014).

In the investigation of conserved Hsf domains, we observed that a class A Hsf (SsuHsf-A9) lacked the AHA motif, which is essential for the transcription activity of Class A Hsf. In tomato, both of the AHA motifs in HsfA1 and HsfA2 have activator potential, and each can be replaced by the other (Döring et al., 2000). A likely reason for our observation is that SsuHsf-A9 exerts its functions by binding to other Class A Hsfs and forming hetero-oligomers.

### *SsuHsf* involvement in developmental processes and stress responses

To survive in different environments, plants have evolved a series of defense strategies against various biotic and/or abiotic stresses (Ahuja et al., 2010). Increasing numbers of studies have reported that Hsfs play pivotal roles in stress tolerance by regulating gene expression (Bharti et al., 2004; Schramm et al., 2006; Giorno et al., 2010; Scharf et al., 2012). *cis*-elements have an essential function in the regulation of gene expression by controlling promoter efficiency (Lescot et al., 2002). Our *in silico* survey of the putative *cis*-elements showed that 20 of the 27 *SsuHsfs* have HSEs in their promoter regions. This implies that these *SsuHsfs* might be regulated by Hsfs themselves (Nover et al., 2001). Additionally, there are two leaf development related *cis*-elements (HD-Zip1 and HD-Zip2) in the promoter of *SsuHsfA7a* (Figure 6), which is consistent with its high expression in leaves (Figure 7).

The *SsuHsfs* were expressed in various tissues. Notably, members in the A1, A8, and B1 subclasses, such as *SsuHsf-A1a, SsuHsf-A1b, SsuHsf-A1c, SsuHsf-A8a, SsuHsf-A8b*, and *SsuHsf-B1*, were constitutively expressed in different tissues. Similar results have been found in *Arabidopsis* and apple. In *Arabidopsis*, Class A1 *Hsfs* are involved in house-keeping processes under normal conditions (Busch et al., 2005). In apple, members in the A1 and B1 subclasses are constitutively expressed in different tissues (Giorno et al., 2012).

Furthermore, the expression data indicated that four of the five duplicated gene pairs exhibited differences in their expression profiles, implying that they may be under different regulation in *S. suchowensis* tissues. Functional diversification of multifamily duplicated genes has been observed in woody species. For example, the *Hsf* and *Hsp* families in *Populus* are clearly divergent in their expression patterns in different tissues and in response to various stress treatments (Zhang et al., 2015). Therefore, the duplicated *SsuHsfs* may have undergone the sub-functionalization for development and/or specific stress conditions.

Studies using tomato and *Arabidopsis* have indicated that *Hsfs* are key regulators in developmental signaling (Schramm et al., 2006; Giorno et al., 2010). HsfA9 plays a unique role during embryogenesis and seed maturation in sunflower and *Arabidopsis* (Almoguera et al., 2002; Kotak et al., 2007). The expression of *AtHsfA9* is regulated by a seed-specific transcription factor, ABSCISIC ACID-INSENSITIVE3, in *Arabidopsis* (Kotak et al., 2007). The interesting role of HsfA9 in seed development might be related with the ABA and auxin signal networks (Carranco et al., 2010). In *S. suchowensis, HsfA9* was specifically expressed in the female catkin (Figure 7) and was induced by ABA treatment (Figure 8), indicating that the HsfA9 protein might have had a conserved function during evolution.

In *Arabidopsis*, AtHsfA1a and AtHsfA1b regulate the early response to heat stress (HS) (Lohmann et al., 2004). The expression of *AtHsfA2* is rapidly induced by HS, and it can enhance and maintain the HSR when the HS is prolonged (Charng et al., 2007). Similarly to AtHsfA2, AtHsfA3 is involved in thermo-tolerance mechanisms (Schramm et al., 2008). In tomato, it was demonstrated that HsfA1a acts as the master regulator of the HSR and cannot be replaced by any other Hsf (Mishra et al., 2002). Although the *Hsf* members in *Arabidopsis* seem to be similar to those in tomato in composition and complexity, no master Hsf has been identified in *Arabidopsis*. The A1-type *SsuHsfs* were expressed at a similar level in leaves from plants growing in control and heat stress conditions, while *SsuHsf-A2* and *SsuHsf-A3* were strongly induced under heat stress conditions (Figure 8). This implies that the two *SsuHsfs* might maintain the HSR.

Compared with Class A *Hsfs*, the members in Class B and C have not been well-studied. The Class B *Hsfs* may act as transcription repressors or co-activators regulating acquired thermotolerance. Some of them form a complex with Class A Hsfs to maintain housekeeping gene expression during the HSR (Bharti et al., 2004). The function of Class C Hsf genes has not yet been fully identified. Notably, the expression of *SsuHsf-B1, -B2a*, -*B2b*, and *-C1* was highly induced in heat, drought, and salt stresses, suggesting that these genes may play important roles in the response to abiotic stresses in *S. suchowensis*.

## Conclusion

In this study, 27 members of the *S. suchowensis Hsf* gene family were identified. Comprehensive analyses of these genes, including phylogeny, gene structure, conserved motifs, and expression profiling in various tissues and under abiotic stresses, were performed. Based on structural characteristics and a comparison of the phylogenetic relationships among the *S. suchowensis, P. trichocarpa*, and *A. thaliana Hsf* families, the 27 SsuHsfs were classified into three classes (A, B, and C). Five gene pairs generated by duplication events were identified in the *SsuHsf* gene family. Expression analyses revealed that they may be involved in developmental processes and abiotic stress responses. This study gives an overview of the *Hsfs* in *S. suchowensis* and provides some insights into the responses of *S. suchowensis* to abiotic stresses, but how *Hsfs* participate in these responses requires further study.

## Author contributions

JZ carried out all the experiments, data analysis and manuscript preparation. YL, HX, JB, and JH helped in data collection, sample preparation and RNA extraction. YL performed most of the RT-PCR experiments. JZ, JJ, and MZ conceived the project, designed the experiments, supervised the analysis and critically revised the manuscript. All authors read and approved the final manuscript.

### Conflict of interest statement

The authors declare that the research was conducted in the absence of any commercial or financial relationships that could be construed as a potential conflict of interest.

## Supplementary material

Table S1 **The complete coding sequences and the corresponding amino acid sequences of *Hsf* genes identified from *S. suchowensis***.

Table S2 **Primer sequences used in experiments**.

Table S3 **Details of the expression data in Figure 8**. The expression data correspond to log2 transformed values compared with control (0h). Data represent the average of three independent experiments ± SE.

Figure S1 **Sequence identity of Hsf proteins in *S. suchowensis*, *P. trichocarpa*, and *A. thaliana***. Amino acid identity among Hsf proteins was analyzed in pairwise fashion.
