# Supplementary figures and images for: The heat shock factor gene family in Salix suchowensis: a genome-wide survey and expression profiling during development and abiotic stresses
Source: Front Plant Sci. 2015 Sep 16;6:748. doi: 10.3389/fpls.2015.00748 (PMC4584977; doi:10.3389/fpls.2015.00748)

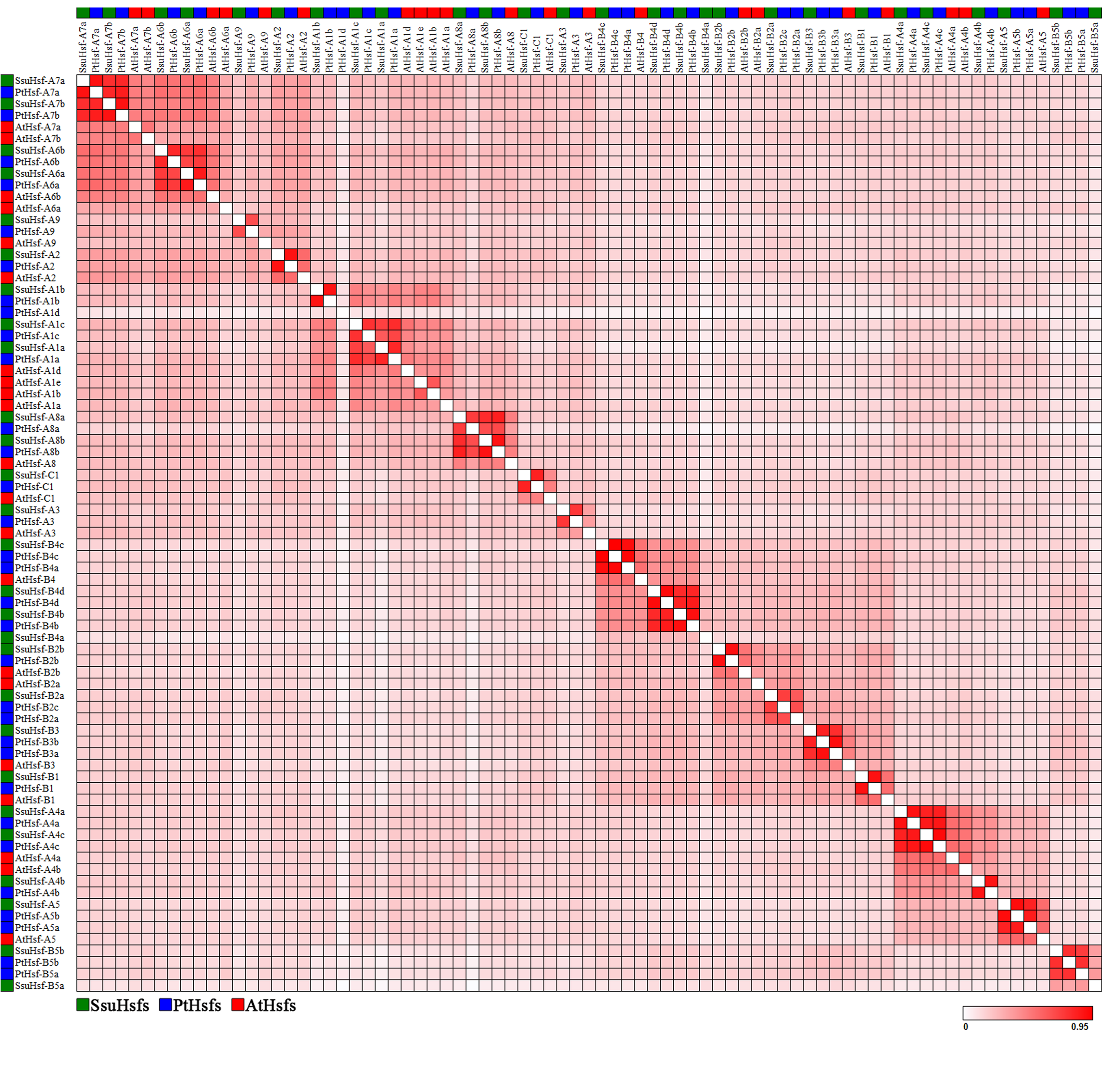

Supplement: Figure S1 — Sequence identity of Hsf proteins in S. suchowensis, P. trichocarpa, and A. thaliana. Amino acid identity among Hsf proteins was analyzed in pairwise fashion. [file Image1.TIF]
